# ECOA-8. Lung adenocarcinoma brain metastasis prediction using tumor DNA methylation profiling

**DOI:** 10.1093/noajnl/vdab070.008

**Published:** 2021-07-05

**Authors:** Jeffrey Zuccato, Yasin Mamatjan, Kenneth Aldape, Gelareh Zadeh

**Affiliations:** 1 Princess Margaret Cancer Centre, Toronto, ON, Canada; 2 National Cancer Institute, Bethesda, MD, USA

## Abstract

**Introduction:**

The development of brain metastases from primary cancer profoundly impacts patient prognosis. Up to one quarter of lung cancers develop brain metastases and subsequent median overall survival is one year. Although clinical factors do not reliably predict brain metastasis development, DNA methylation signatures have been recently shown to predict outcomes in other cancers. It is hypothesized that DNA methylation signatures predicting brain metastasis development from lung cancer will be identified. This work may allow for treatment strategies that prevent brain metastasis development in high risk lung cancer patients.

**Methods:**

DNA methylation profiling was undertaken on N=124 lung adenocarcinoma patients. In a randomly selected 70% training cohort, differentially methylated CpG sites between patients developing and not developing brain metastases were identified and used to build a generalized boosted regression model. Patients in the independent 30% testing cohort were assigned brain metastasis risk scores by the model.

**Results:**

Brain metastases developed in 49/124 (39.5%) of patients and 2.3K CpG sites were significantly differentially methylated between patients developing and not developing metastases. Methylation-based brain metastasis risk scores predicted time to brain metastasis development in the testing cohort (Univariate cox: HR=3.2, 95% CI 1.1–9.4, p=0.03). A multivariate cox analysis assessing tumor size and nodal positivity together with methylation scores as covariates identified methylation scores as the only independent predictor of brain metastasis development in the testing cohort (HR=4.3, 95%CI 1.1–17, p=0.038).

**Conclusions:**

DNA methylation signatures in lung adenocarcinomas predict brain metastasis development independently from the non-metastatic components of cancer stage. Future work developing a comprehensive nomogram utilizing methylation scores together with clinical factors to determine patient specific risk values may aid in treatment decisions and patient prognosis counselling.

